# Measuring depression in nursing home residents with the MDS and GDS: an observational psychometric study

**DOI:** 10.1186/1471-2318-5-1

**Published:** 2005-01-01

**Authors:** Melissa Koehler, Terry Rabinowitz, John Hirdes, Michael Stones, G Iain Carpenter, Brant E Fries, John N Morris, Richard N Jones

**Affiliations:** 1 New Hanover Regional Medical Center, 2131 S 17th Street, Wilmington, NC 28401, USA; 2 University of Vermont College of Medicine, Fletcher Allen Health Care, 111 Colchester Avenue, Burlington, VT 05401-1473, USA; 3 Department of Health Studies and Gerontology, University of Waterloo, Waterloo, Ontario N2L 3G1, Canada; 4 Department of Psychology, Lakehead University, Thunder Bay, Ontario, Canada; 5 The University of Kent, Canterbury, Center for Health Service Studies, George Allen Wing, Kent, CT2 7NF, UK; 6 University of Michigan Institute of Gerontology, 300 North Ingalls Ann Arbor, MI 48109-2007, USA; 7 Research and Training Institute, Hebrew Rehabilitation Center for Aged, Boston, 1200 Centre Street, Massachusetts 02131 USA; 8 Division of Gerontology, Beth Israel Deaconess Medical Center, Division on Aging, Harvard Medical School, Boston, MA USA

## Abstract

**Background:**

The objective of this study was to examine the Minimum Data Set (MDS) and Geriatric Depression Scale (GDS) as measures of depression among nursing home residents.

**Methods:**

The data for this study were baseline, pre-intervention assessment data from a research study involving nine nursing homes and 704 residents in Massachusetts. Trained research nurses assessed residents using the MDS and the GDS 15-item version. Demographic, psychiatric, and cognitive data were obtained using the MDS. Level of depression was operationalized as: (1) a sum of the MDS Depression items; (2) the MDS Depression Rating Scale; (3) the 15-item GDS; and (4) the five-item GDS. We compared missing data, floor effects, means, internal consistency reliability, scale score correlation, and ability to identify residents with conspicuous depression (chart diagnosis or use of antidepressant) across cognitive impairment strata.

**Results:**

The GDS and MDS Depression scales were uncorrelated. Nevertheless, both MDS and GDS measures demonstrated adequate internal consistency reliability. The MDS suggested greater depression among those with cognitive impairment, whereas the GDS suggested a more severe depression among those with better cognitive functioning. The GDS was limited by missing data; the DRS by a larger floor effect. The DRS was more strongly correlated with conspicuous depression, but only among those with cognitive impairment.

**Conclusions:**

The MDS Depression items and GDS identify different elements of depression. This may be due to differences in the manifest symptom content and/or the self-report nature of the GDS versus the observer-rated MDS. Our findings suggest that the GDS and the MDS are not interchangeable measures of depression.

## Background

Depression is common among residents of nursing homes [1]. Of the many instruments used to identify depression in the elderly, the Geriatric Depression Scale (GDS)[2] is probably the most widely used in research settings. The original version comprises 30 items, whereas subsequent versions have been proposed with 15, 12 and later five items [3-5]. None of the items query somatic complaints, rather, questions inquire about the respondent's perspective on their life over the previous week.

The Minimum Data Set (MDS), created in response to the US Omnibus Budget Reconciliation Act of 1987, aims to provide a uniform, standardized, and comprehensive assessment of residents in nursing homes [6]. The MDS has undergone several revisions since its inception, and is currently undergoing another revision process. Section E of MDS version 2.0 assesses 16 depression symptoms that capture verbal and non-verbal indicators of depressed mood or anxiety as perceived by nursing home staff. A summary scale, the Depression Rating Scale (DRS), uses a subset of seven of these symptoms and has been shown to be a reliable and valid measure of depression among nursing home residents [7]. The MDS DRS is therefore, by nature of its ubiquity, potentially the most widely available depression assessment instrument for older adults in nursing home settings.

This study compares the measurement properties of two measures of geriatric depression: the GDS and the MDS. The GDS is evaluated via a 15 and five item form. The MDS is evaluated by the items located in Section E in total and as a subset included in the Depression Rating Scale.

## Methods

Participants were nursing home residents living in nine Massachusetts nursing facilities participating in a research study between July 1994 and March 1998. Details of the study are described elsewhere [8]. All data used in this study reflect baseline and pre-intervention observations. The sample is considered representative of persons living in nursing homes in Eastern Massachusetts. Potential participants consented individually or by proxy to participate in a research study, using a protocol approved by the local Institutional Review Board. Exclusion criteria included: 1) a terminal prognosis, 2) a projected stay of less than 90 days, or 3) health complications that prohibited contact. Seven hundred four (n = 704) residents and/or their proxies participated in the baseline assessment, representing 67% of those eligible (14% of screened residents were ineligible). Seventy-seven percent of residents were women, 95% were White, non-Hispanic, and the median age was 86 years (interquartile range, 79–91 years). About half (54%) of residents had a chart diagnosis of dementia.

### Assessment of depression

Residents were evaluated with the Minimum Data Set (MDS) Resident Assessment Instrument version 2.0 [6] as well as a 15-item version of the Geriatric Depression Scale (GDS-15) [2,3,9]. Trained research nurses collected the observations. For this analysis, we also considered a 5-item version of the GDS (GDS-5) [5]. MDS data collected and used in this analysis included demographic and clinical characteristics, level of cognitive and communicative functioning, and symptoms of depression. We used a simple sum of all symptoms in MDS section E1, referred to as E1SUM, as one MDS-based measure of depression. We also used a subset of seven of these MDS symptoms to code the MDS Depression Rating Scale (DRS) [7].

We also examined the depression symptom scales with regard to indicators of clinically recognized depression: chart diagnoses of depression and recent use of antidepressant medication. These data were also obtained by research nurses using structured chart review forms keyed to data elements collected with the MDS. MDS assessors are instructed to note the presence of a disorder related to the resident's current functional, cognitive, and behavioural status, medical treatments, and risk of death [10]. Among the disorders assessed is depression. The MDS manual is not specific with regard to clinical or diagnostic criteria for indicating depression diagnoses. The MDS also includes assessment of psychotropic medication use in the seven days preceding the assessment, including antidepressant use. In our analyses, we compared residents receiving any antidepressant with those receiving no antidepressant.

### Assessment of cognitive impairment

We stratified the sample into two groups based on the severity of cognitive impairment. The impaired group comprised residents who were comatose (MDS 2.0 item B1 = 1), and/or with a short-term memory problem (B2a = 1) and those who only rarely/never make themselves understood (C4 = 3). All other residents were assigned to the "cognitively intact" group. This decision rule matches a screening rule for the MDS versus GDS depression symptom assessment proposed in the US Centers for Medicare & Medicaid Services' (CMS) working draft of the MDS version 3.0 [11].

### Analytic approach

We compared sample statistics and psychometric properties for each of the four depression scales across strata defined by cognitive impairment. We evaluated missing data by examining the proportion of residents with complete data on all assessment items, and also by the proportion of residents with complete data on a majority of items in the scale. For all other analyses we substituted missing values with the mean of the resident's non-missing items if a majority of the scale items were not missing. We examined means, standard deviations (SD), proportion of residents scoring at the floor, the internal consistency reliability (coefficient alpha [12], examined with and without a row-wise mean substitution rule for missing item responses) and the correlation among the depression scales.

Finally, we examined the relationship of the scale scores to clinical indicators of depression: chart diagnoses of depression and a record of antidepressant use. We compared the mean of the scale scores across each of four cells formed by crossing antidepressant use and depression diagnoses. These comparisons were also made within cognitive impairment strata. Within strata, cell means were standardized with respect to the mean and standard deviation of the group of residents that neither received antidepressants nor had a chart diagnosis of depression. In this way, cell mean differences can be interpreted on an effect size metric [13]. All analyses were conducted with STATA software (College Station, Texas).

## Results

### Sample statistics and missing data

Table 1 presents the sample statistics and psychometric properties for the comparison of the GDS and the MDS depression assessment instruments, stratified by level of cognitive impairment. Approximately 70% of residents were classified as cognitively impaired. Among the cognitively impaired, a majority had missing values for the GDS-derived scales (i.e., the GDS-15 and the GDS-5). A substantial proportion (about one in six) of residents were missing data on all GDS items. However, for the GDS-5, about 1 in 20 of the residents without cognitive impairment were missing data on all GDS items. On the other hand, the presence of missing data on the MDS-derived scales (i.e., E1SUM and DRS) was essentially nil in both cognitive impairment groups. About a third of residents had no missing values on the GDS-15 and more than half had missing data on the GDS-5.

**Table 1:** Sample statistics and psychometric properties of Geriatric Depression Scale and MDS Depression Rating Scale.

Sample statistic or psychometric property	Cognitively Impaired Group (n = 495)	Cognitively Intact Group (n = 209)	All Participants (N = 704)
Number (%) with missing data on all items			
GDS-15	81 (16%)	35 (17%)	116 (16%)
GDS-5	110 (22%)	5 (2%)	115 (16%)*
E1SUM	3 (1%)	2 (1%)	5 (1%)
DRS	0 (0%)	0 (0%)	0 (0%)
Number (%) with complete data on all items			
GDS-15	139 (28%)	111 (53%)	250 (36%)*
GDS-5	223 (45%)	157 (75%)	380 (54%)*
E1SUM	492 (99%)	207 (99%)	699 (99%)
DRS	494 (100%)	209 (100%)	703 (100%)
Number (%) with majority of scale items not missing			
GDS-15	357 (72%)	203 (97%)	560 (80%)*
GDS-5	352 (71%)	200 (96%)	552 (78%)*
E1SUM	495 (100%)	209 (100%)	704 (100%)
DRS	495 (100%)	209 (100%)	704 (100%)
Mean* (SD)			
GDS-15	4.7 (3.5)	4.9 (3.4)	4.8 (3.5)
GDS-5	2.0 (1.5)	2.1 (1.6)	1.5 (1.6)
E1SUM	3.9 (3.8)	2.7 (3.4)	3.6 (3.7)*
DRS	1.9 (2.1)	1.8 (2.3)	1.9 (2.2)
Proportion at floor			
GDS-15	0.081	0.053	0.072
GDS-5	0.166	0.167	0.166
E1SUM	0.206	0.349	0.249*
DRS	0.356	0.397	0.368
Alpha – internal consistency reliability – row-wise complete cases only			
GDS-15	0.799	0.781	0.791
GDS-5	0.609	0.597	0.602
E1SUM	0.695	0.738	0.708
DRS	0.542	0.672	0.583*
Alpha – internal consistency reliability – row-wise mean substitution for missing data^†^			
GDS-15	0.825	0.798	0.814
GDS-5	0.663	0.634	0.651
E1SUM	0.695	0.738	0.708
DRS	0.542	0.672	0.583*
Correlation coefficients			
(GDS-5, GDS-15)	0.858	0.852	0.856
(DRS, GDS-15)	0.073	0.098	0.080
(DRS, GDS-5)	0.065	0.064	0.062
(E1SUM, GDS-15)	0.068	0.096	0.072
(E1SUM, GDS-5)	0.055	0.058	0.049
(E1SUM, DRS)	0.850	0.940	0.865*

Abbreviations: GDS-15, 15-item Geriatric Depression Scale; GDS-5, 5-item Geriatric Depression Scale; DRS, Minimum Data Set (MDS) Depression Rating Scale; E1SUM denotes the sum of all mood and anxiety items in section E1 of the MDS; SD, standard deviation

* For each respondent, if a majority of the items were not missing, any missing item was replaced with the mean of the non-missing items.

† Missing item responses replaced with mean score for all respondents 'non-missing' on that item.

On the other hand, about half of the cognitively intact group had no missing GDS-15 scores and three-quarters had no missing GDS-5 scores. Using an item-level missing data mean substitution rule, predicated on a resident having at least a majority of items present, the frequency of missing data for scale scores diminished for the GDS-derived scales. However, the impact of missing data remains an important problem for GDS-derived measures among the cognitively impaired: between one quarter and one third of the cognitively impaired still had missing data. All noted differences in the frequency of missing data describe large effect sizes (in Cohen's effect size taxonomy [13]) and are statistically significant (P < .001).

The very high frequency of missing data for the GDS encouraged us to examine the frequency of missing data at the item level. We present this information in Table 2, limiting the sample to those missing at least one but not all GDS items. The item with the greatest frequency of missing data was item 15 ("do you think that most people are better off than you are") for both the cognitively impaired group and those with better cognitive functioning. The item with the fewest missing values was item 5 among the cognitively impaired group ("are you in good spirits most of the time?") and item 1 among those without cognitive impairment ("are you basically satisfied with your life?"). Although the proportion with missing data on each item differed significantly across cognitive impairment strata for only one item (item 9), many of the differences across groups depict medium or larger effect sizes (items 2, 9, 10, 12).

**Table 2:** Proportion of residents with missing data on individual items of the Geriatric Depression Scale. Limited to residents missing at least one but less than all 15 items.

Item descriptions	Cognitively Impaired Group (n = 251)	Cognitively Intact Group (n = 93)	All Participants (N = 344)
1. Are you basically satisfied with your life?*	37 (15%)	6 (6%)	43 (13%)
2. Have you dropped many of your activities and interests?	68 (27%)	10 (11%)	78 (23%)
3. Do you feel that your life is empty?	57 (23%)	16 (17%)	73 (21%)
4. Do you often get bored?*	47 (19%)	14 (15%)	61 (18%)
5. Are you in good spirits most of the time?	30 (12%)	9 (10%)	39 (11%)
6. Are you afraid that something bad is going to happen?	44 (18%)	13 (14%)	57 (17%)
7. Do you feel happy most of the time?	35 (14%)	12 (13%)	47 (14%)
8. Do you often feel helpless?*	55 (22%)	20 (22%)	75 (22%)
9. Do you prefer to stay in your room, rather than go out and doing new things?*	88 (35%)	11 (12%)	99 (29%)
10. Do you feel you have more problems with memory than most?	80 (32%)	11 (12%)	91 (26%)
11. Do you think that it is wonderful to be alive?	48 (19%)	14 (15%)	62 (18%)
12. Do you feel pretty worthless they way you are now?*	78 (31%)	14 (15%)	92 (27%)
13. Do you feel full of energy?	64 (25%)	14 (15%)	78 (23%)
14. Do you feel that your situation is hopeless?	74 (29%)	16 (17%)	90 (26%)
15. Do you think that most people are better off than you?	110 (44%)	31 (33%)	141 (41%)

* included in GDS-5

### Mean level of depression

The means for the GDS- and MDS-derived scales were different between the cognitively intact and impaired groups (Table 1). Of note, the cognitively impaired group received higher scores on both the GDS-15 and GDS-5, indicating higher levels of depression. While these differences were small [13] they describe statistically significant differences (P < .05). Conversely, the cognitively intact residents within both the DRS and E1SUM had higher depression ratings. The difference between cognitive impairment groups was trivial[13] and not statistically different for the GDS (P = .79), but of moderate size[13] and statistically significant for E1SUM (P < .001).

### Floor effect

**Figure 1:**
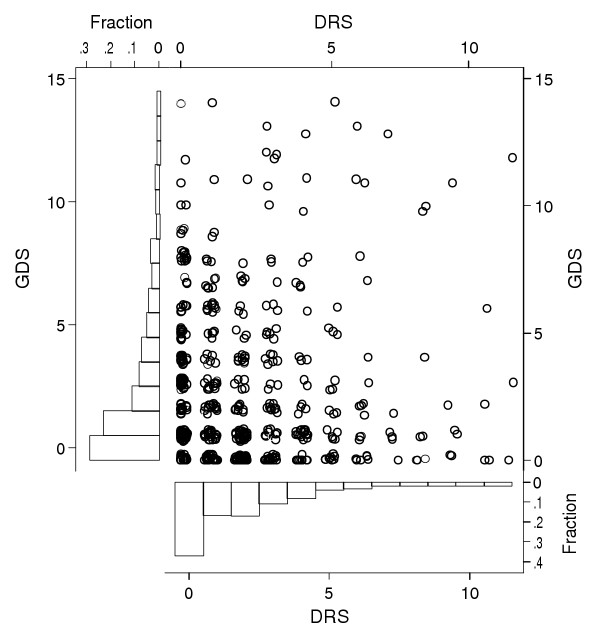
Scatter plot of 15-item Geriatric Depression Scale (GDS) and Depression Rating Scale (DRS) scores among 560 Nursing Home Residents in Nine Massachusetts Nursing Homes.

Figure 1 illustrates several characteristics of the GDS-15 and the DRS, including the floor effect. Both instruments produce distributions with a high proportion of respondents scoring zero. For the GDS-derived measures as well as the DRS, the proportion scoring at the floor is similar for both cognitive impairment groups. The difference in the proportion scoring at the floor on the E1SUM measure is moderately different between the cognitively intact and cognitively impaired groups (P < 0.01).

### Internal consistency reliability

The GDS-15 had the highest internal consistency reliability coefficient, and the lowest was observed for the DRS. However, this difference does not substantially exceed that expected due to the greater scale length of the GDS-15. Using the Spearman-Brown prophecy formula [14], the reliability of the DRS for the total sample would be 0.72 if it had 15 similar items (instead of 7), which is closer to that observed for the GDS-15 (for residents with complete data). There was no statistically significant difference in the internal consistency reliability across cognitive impairment groups (using the variance ratio test[15]) for the GDS-derived scales and the E1SUM. However, the differences across cognitive impairment strata for the DRS were statistically significant (P < .01). We also note that the estimated internal consistency reliability can be artificially inflated by using a row-wise (i.e., person-wise) mean substitution rule for missing data. This artificial inflation affects GDS-derived scales but not DRS-derived scales.

### Correlation of DRS and GDS

The correlation between the GDS- and DRS- derived measures were not statistically different from zero. All of the differences in correlation coefficients are similar among the cognitively impaired and cognitively intact and not statistically different, except for the correlation of the E1SUM and DRS (P < .001).

### Relation of DRS and GDS to indicators of conspicuous depression

The comparison of mean depression symptom scale scores among residents that received antidepressants and/or had a chart diagnosis of depression, within and across cognitive impairment strata, is reported in Table 3. For the GDS-15, within both cognitive impairment groups, there are trivial marginal differences in the mean score for either a depression diagnosis or record of antidepressant use. For the GDS-5, the mean score is somewhat higher for residents with a diagnosis or record of antidepressant use. These differences correspond to small to moderate effect size differences [13]. The marginal differences are not statistically significant within cognitive impairment strata, but for the total sample the pattern of means are essentially the same and are statistically significant for a depression diagnosis (P = .02) and approach significance for antidepressant use (P = .05).

**Table 3:** Mean depression scale score as a function of the presence of diagnosis of depression or receiving antidepressants. Means are standardized to the mean and variance of the scale score for the group without a diagnosis and who did not receive an antidepressant.

	Cognitively Impaired Group (N = 495)				Cognitively Intact Group (N = 209)			Total (N = 704)		
			
		Depression Diagnosis			Depression Diagnosis			Depression Diagnosis		
**GDS-15**		No	Yes	Total	No	Yes	Total	No	Yes	Total
Antidepressant Use	No	0.00	0.14	0.02	0.00	.47	0.06	0.00	0.26	0.03
		(203)	(26)	(229)	(116)	(16)	(132)	(319)	(42)	(361)
			
	Yes	0.07	0.43	0.31	0.29	0.36	0.09	0.16	0.41	0.32
		(42)	(86)	(128)	(30)	(41)	(71)	(72)	(127)	(199)
			
	Total	0.01	0.36	0.12	0.06	0.39	0.15	0.03	0.37	0.13
		(245)	(112)	(357)	(146)	(57)	(203)	(391)	(169)	(560)
**GDS-5**										
Antidepressant Use	No	0.00	0.05	0.01	0.00	0.51	0.06	0.00	0.23	0.03
		(199)	(23)	(222)	(115)	(16)	(131)	(314)	(39)	(353)
			
	Yes	0.00	0.31	0.21	0.23	0.28	0.26	0.09	0.30	0.22
		(44)	(86)	(130)	(30)	(39)	(69)	(74)	(125)	(199)
			
	Total	0.00	0.26	0.08	0.05	0.35	0.13	0.02	0.29	0.10
		(243)	(109)	(352)	(145)	(55)	(200)	(388)	(164)	(552)
**E1SUM**										
Antidepressant Use	No	0.00	0.43	0.05	0.00	0.23	0.03	0.00	0.37	0.05
		(295)	(39)	(334)	(119)	(18)	(137)	(414)	(57)	(471)
		
	Yes	0.60	0.55	0.57	0.48	-.06	0.18	0.55	0.37	0.44
		(62)	(99)	(161)	(31)	(41)	(72)	(93)	(140)	(233)
			
	Total	0.11	0.52	0.22	0.10	0.04	0.08	0.10	0.37	0.18
		(357)	(138)	(495)	(150)	(59)	(209)	(507)	(197)	(704)
**DRS**										
Antidepressant Use	No	0.00	0.40	0.05	0.00	0.16	0.02	0.00	0.32	0.04
		(294)	(39)	(333)	(119)	(18)	(137)	(413)	(57)	(470)
			
	Yes	0.59	0.59	0.59	0.51	-0.15	0.13	0.56	0.33	0.42
		(62)	(99)	(161)	(31)	(41)	(72)	(93)	(140)	(233)
			
	Total	0.10	0.54	0.22	0.11	-0.06	0.06	0.10	0.33	0.17
		(356)	(138)	(494)	(150)	(59)	(209)	(506)	(197)	(703)

Abbreviations: GDS-15, 15-item Geriatric Depression Scale; GDS-5, 5-item Geriatric Depression Scale; DRS, Minimum Data Set (MDS) Depression Rating Scale; E1SUM denotes the sum of all mood and anxiety items in section E1 of the MDS

For the MDS-derived depression scales, the differences in means associated with antidepressant use and depression diagnoses are much more dramatic than for the GDS-derived scales, but only among the cognitively impaired. For both the E1SUM and DRS, the marginal differences for depression diagnoses and antidepressant use describe moderate to large effect sizes. These differences are statistically significant (both P < .001). On the other hand, among those residents not identified as cognitively impaired, the marginal differences associated with antidepressant use and a depression diagnosis were trivial to small and not statistically significant.

## Discussion

In this study, we found that MDS- and GDS-derived depression measures were not correlated with one another, but were apparently adequately reliable measures of their intended construct. Thus, we infer that the MDS and the GDS measure different aspects of nursing home residents' depression. Each scale has specific strengths and limitations. The practical utility of the GDS is undermined by a very high frequency of missing data. The proportion of GDS responses missing differs greatly by level of cognitive functioning. The floor effect limits both instruments. While the internal consistency reliability is apparently greater for the GDS, this may simply be due to the greater number of items on the GDS. We observed a weak relationship between GDS-5 scale scores and clinical indicators of depression (diagnoses, antidepressant use), but the strong association between MDS-derived scales and clinical indicators was only seen among cognitively impaired residents.

Other investigators have found a low correlation between the DRS and the GDS and other instruments for assessing depression, but these findings vary according to data collection strategies. For example, Anderson et al found a low correlation between the MDS DRS abstracted from residents' charts and symptom data collected with the GDS (r = .13) and the Hamilton Depression Rating Scale (HDRS; r = 0.24) using research interviewers [16]. Similarly, Hendrix et al[17] found a lack of correspondence between MDS depression symptoms and depression classified using a cut-point on the Cornell Scale for Depression in Dementia (CSDD). Hendrix and colleagues attributed the low agreement of CSDD and MDS to different data collection strategies. In their study, the CSDD was collected by primary caregivers, while the MDS was abstracted from the chart, and these authors suggest that the nurse administrators that completed the MDS did not consult the primary caregivers and the resident in completing the MDS depression items. Contrast with these findings a recent study by Ruckdeschel and colleagues [18], who converted the MDS items into a self-report assessment device and reported a very high correlation with depression symptom data collected with the GDS (r = 0.71).

In our study, the assessment methods followed more closely how they were designed to be used. The GDS was used as a direct interview, and the MDS was used as instructed in the MDS manual [6], and included review of the chart, semi-structured interview with the resident, direct caregivers, and available family members or key informants, in order to arrive at final symptom ratings. MDS- and GDS-derived measures were comparably reliable after adjustments for test length, but were nevertheless not very highly correlated. Therefore, whatever differentiates the dimensions assessed by the two devices, it is probably an influence beyond the level of assessor training and the rigor of the evaluation.

The fact that the MDS- and GDS-derived scales do not correlate implies that the two scales evaluate different aspects of a resident's depression. For positive MDS depression symptom ratings, residents must visibly act by making negative statements, be easily angered, and display unrealistic fears to trigger MDS symptoms. The GDS asks residents if they are satisfied with their life, feel helpless or worthless, and are often bored. It is conceivable that GDS-derived measures capture a brooding mental set, reflective of a dysphoric personality trait or adjustment disorder (for example in response to a recent change in living situation) rather than the presence of major depression.

The lack of correlation between MDS depression measures has implications for proposed revisions to the MDS. Until more is known about the phenomenology and clinical validity of syndromes measured by these and other depression measures used in long-term care settings, adding self-report of symptoms of depression to the MDS is supported by our findings. We note that both CMS's draft revision of the MDS[11] and new versions of assessment instruments developed for other care settings by inter*RAI *include a provision for self-assessment of depression [19]. However, our findings may have further implications for CMS's revision of the MDS. The current draft of the revision calls for a sub-set of MDS section E items for those who are cognitively impaired, and direct questioning with the GDS-5 for those who are cognitively intact. Our findings suggest a more conservative approach might be to use both for all residents, or the MDS for all residents and the GDS for all residents who can communicate regardless of cognitive level. Two key findings underlie this recommendation. First, we find that the GDS and MDS are not complementary, but orthogonal. Second, we find no evidence for compromised measurement properties of the GDS among those with cognitive impairment.

## Conclusions

The Geriatric Depression Scale and Minimum Data Set mood items measure different aspects of the depression syndrome among nursing home residents. The two measures cannot be treated as exchangeable or equivalent, and each has it's own strengths and limitations. The GDS uses self-report, but as a consequence suffers from a high frequency of missing data. The MDS relies upon informant ratings and therefore provides information about most residents. Although the GDS has higher internal consistency reliability than MDS, this is not beyond that expected due to greater scale length. The MDS measures were more strongly related to antidepressant use and record of a diagnosis of depression than were GDS measures. These results highlight the fact that more psychometric research is needed to better understand and improve the measurement of depression among frail nursing home residents.

## List of abbreviations used

GDS Geriatric Depression Scale

MDS Minimum Data Set

DRS Depression Rating Scale

CSDD Cornell Scale for Depression in Dementia

HDRS Hamilton Depression Rating Scale

## Competing interests

No financial competing interests. TR, JH, GIC, BEF and JNM are fellows of InterRAI, a non-profit group dedicated to improving the health care for persons who are elderly, frail or disabled. InterRAI owns the copyright to the MDS for nursing homes and many other care settings. InterRAI offers free licenses for the use of assessment forms of which it owns the copyright to. Therefore, there are no financial competing interests for members of the writing group. For more information please visit .

## Authors' contributions

MK and RNJ conceived of the study. MK drafted the manuscript. RNJ performed the analyses and provided critical review of and completed the manuscript. JNM obtained the data, participated in the analysis and provided critical review of the manuscript. JH, MS, GIC and BEF provided critical review of the analyses and manuscript. All authors read and approved the final manuscript.

## Pre-publication history

The pre-publication history for this paper can be accessed here:



## References

[B1] Jones RN, Marcantonio EM, Rabinowitz T (2003). Prevalence and correlates of recognized depression in U.S. nursing homes. J Am Geriatr Soc.

[B2] Yesavage JA, Brink TL, Rose TL, Lum O, Huang V, Adey M, Leirer VO (1982). Development and validation of a geriatric depression screening scale: a preliminary report. J Psychiatr Res.

[B3] Sheikh JI, Yesavage J (1986). Geriatric Depression Scale (GDS): Recent evidence and development of a shorter version. Clin Gerontol.

[B4] Sutcliffe C, Cordingley L, Burns A, Mozley CG, Bagley H, Huxley P, Challis D (2000). A new version of the geriatric depression scale for nursing and residential home populations: the geriatric depression scale (residential) (GDS-12R). Int Psychogeriatr.

[B5] Hoyl MT, Alessi CA, Harker JO, Josephson KR, Pietruszka FM, Koelfgen M, Mervis JR, Fitten LJ, Rubenstein LZ (1999). Development and testing of a five-item version of the Geriatric Depression Scale. J Am Geriatr Soc.

[B6] Morris JN, Bernabei R, Ikegami N, Gilgen R, Frijters D, Hirdes JP, Fries BE, Steel K, Carpenter I, DuPasquier JN, Henrard JC (1999). RAI-Home Care (RAI-HC)(C) Assessment Manual for Version 2.0.

[B7] Burrows AB, Morris JN, Simon SE, Hirdes JP, Phillips C (2000). Development of a minimum data set-based depression rating scale for use in nursing homes. Age Ageing.

[B8] Morris JN, Fiatarone M, Kiely DK, Belleville-Taylor P, Murphy K, Littlehale S, Ooi WL, O'Neill E, Doyle N (1999). Nursing rehabilitation and exercise strategies in the nursing home. J Gerontol A Biol Sci Med Sci.

[B9] Yesavage JA (1988). Geriatric Depression Scale. Psychopharmacol Bull.

[B10] Health Care Financing Administration (1995). Long Term Care Resident Assessment Instrument User's Manual Version 2.0.

[B11] Centers for Medicare and Medicaid Services Draft Version of MDS 3.0 (The Draft MDS 3.0 is a CMS working document). http://www.cms.hhs.gov/quality/mds30/DraftMDS30.pdf.

[B12] Cronbach LJ (1951). Coefficient alpha and the internal structure of tests. Psychometrika.

[B13] Cohen J (1988). Statistical power analysis for the behavioral sciences.

[B14] Guilford JP (1936). Psychometric Methods.

[B15] Berk RA (1982). Handbook of methods for detecting test bias.

[B16] Anderson RL, Buckwalter KC, Buchanan RJ, Maas ML, Imhof SL (2003). Validity and reliability of the Minimum Data Set Depression Rating Scale (MDSDRS) for older adults in nursing homes. Age Ageing.

[B17] Hendrix CC, Sakauye KM, Karabatsos G, Daigle D (2003). The use of the Minimum Data Set to identify depression in the elderly. J Am Med Dir Assoc.

[B18] Ruckdeschel K, Thompson R, Datto CJ, Streim JE, Katz IR (2004). Using the Minimum Data Set 2.0 Mood Disturbance Items as a Self-Report Screening Instrument for Depression in Nursing Home Residents. Am J Geriatr Psychiatry.

[B19] InterRAI Corporation (2002). MDS/RAI Assessment Instruments. WWW Page Accessed September 25, 2002 Last Update August 15, 2001 http://wwwinterraiorg.

